# Expression of Human Paraoxonase 1 Decreases Superoxide Levels and Alters Bacterial Colonization in the Gut of *Drosophila melanogaster*

**DOI:** 10.1371/journal.pone.0043777

**Published:** 2012-08-30

**Authors:** Alejandro A. Pezzulo, Emma E. Hornick, Michael V. Rector, Miriam Estin, Anna C. Reisetter, Peter J. Taft, Stephen C. Butcher, A. Brent Carter, J. Robert Manak, David A. Stoltz, Joseph Zabner

**Affiliations:** 1 Department of Internal Medicine, Carver College of Medicine, University of Iowa, Iowa City, Iowa, United States of America; 2 Department of Pediatrics, Carver College of Medicine, University of Iowa, Iowa City, Iowa, United States of America; 3 Department of Biology, University of Iowa, Iowa City, Iowa, United States of America; 4 Free Radical and Radiation Biology Program, University of Iowa, Iowa City, Iowa, United States of America; Queen Mary University of London, United Kingdom

## Abstract

Paraoxonases (PON) are a family of proteins (PON1, 2 and 3) with multiple enzymatic activities. PON1 interferes with homoserine lactone-mediated quorum sensing in bacteria and with reactive oxygen species (ROS) in humans and mice. *PON1* gene mutations have been linked to multiple traits, including aging, and diseases of the cardiovascular, nervous and gastrointestinal system. The overlapping enzymatic activities in the PON family members and high linkage disequilibrium rates within their polymorphisms confound animal and human studies of PON1 function. In contrast, arthropods such as *Drosophila melanogaster* have no PON homologs, resulting in an ideal model to study interactions between PON genotype and host phenotypes. We hypothesized that expression of PON1 in *D. melanogaster* would alter ROS. We found that PON1 alters expression of multiple oxidative stress genes and decreases superoxide anion levels in normal and germ-free *D. melanogaster*. We also found differences in the composition of the gut microbiota, with a remarkable increase in levels of *Lactobacillus plantarum* and associated changes in expression of antimicrobial and cuticle-related genes. PON1 expression directly decreased superoxide anion levels and altered bacterial colonization of the gut and its gene expression profile, highlighting the complex nature of the interaction between host genotype and gut microbiota. We speculate that the interaction between some genotypes and human diseases may be mediated by the presence of certain gut bacteria that can induce specific immune responses in the gut and other host tissues.

## Introduction

The human paraoxonases (PON1, PON2 and PON3) are proteins with promiscuous enzymatic activities and with distinct tissue expression profiles [1]–[6]. Members of this gene family exhibit phosphotriesterase, esterase and lactonase activity to varying degrees [1], [7]–[9]; of these, PON1 has been the most extensively studied. PON1 was originally named for its capacity to hydrolyze paraoxon and other organophosphates [10], and has since been associated to an astounding number of traits and diseases affecting multiple organ systems [2]. While the phosphotriesterase function of PON1 is responsible for its organophosphate-degrading capacity, structure-activity studies suggest that lactonase activity is its native function [9].

Mutations in the human *PON1* gene have been associated with aging and diseases of the cardiovascular, nervous, endocrine and gastrointestinal systems [2], [11], [12]. A possible mechanism for these phenotypes may be related to HDL-dependent and independent antioxidant properties of PON1 [13], [14] and its effects on levels of hydroperoxides and platelet activating factor, which may also affect oxidative stress in tissues [15]–[18]. PON1 can also hydrolyze acyl-homoserine lactones used by quorum-sensing bacteria to regulate multiple virulence factors during colonization of new environments [19]–[22]. This could at least indirectly explain the association of mutations in *PON1* with Crohn's disease and ulcerative colitis, or perhaps other phenotypes associated with the gut microbiota such as obesity and cardiovascular disease [23]–[29].

Describing how mutations in *PON1* result in the associated phenotypes remains an elusive goal for multiple reasons. Mutations in each of the three *PON* family members are in strong linkage disequilibrium with mutations in other *PON* genes or with different genes [30]–[32]. A phenotype associated to genetic variation in *PON1* may therefore be caused by a mutation in another gene. Moreover, the close proximity of the genomic loci for *PON1*, *PON2 and PON3* results in recombination rates that makes generation of triple KO mice difficult and, in single knockout mice, lack of one *PON* member may be compensated by the remaining two members [21].

Although paraoxonases are conserved across a wide range of species, arthropods (including the versatile *Drosophila melanogaster*) lack a homolog [20], [22]. Therefore, we have previously used *D. melanogaster* as a model to study paraoxonases in the absence of proteins with overlapping enzymatic activity that can confound analyses of genotype-phenotype interactions [22].

In the *Drosophila* midgut, reactive oxygen species generated and controlled by the duox system [33]–[37] control intestinal bacterial populations and suppress virulent pathogens such as *Erwinia carotovora* and *Pseudomonas entomophila* from invading the host. Activation of the Toll and Imd pathways to regulate multiple antimicrobial peptides provides an additional mechanism allowing a pathogen class-specific immune response [35]–[37]. Whether and how these mechanisms directly regulate symbiotic bacteria in the normal gut remains a less clear but distinct possibility [38]–[41].

Here, we hypothesized that expression of human PON1 in *D. melanogaster* would directly result in altered levels of reactive oxygen species in the gut. Moreover, we hypothesized that abnormal reactive oxygen species levels would affect the composition of gut symbionts.

## Results

### Expression of human PON1 alters expression of oxidative stress genes in *D. melanogaster*

**Figure 1:**
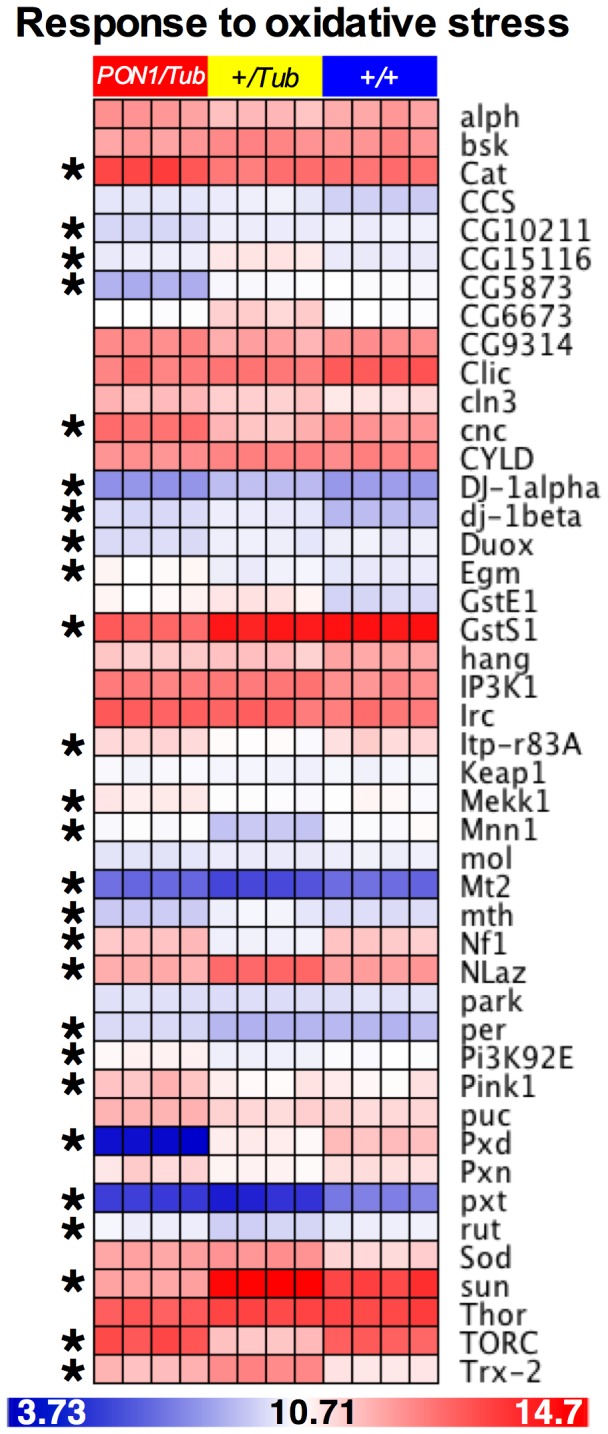
Expression of human PON1 alters expression of oxidative stress genes in *D. melanogaster*. Heatmap of expression values of genes in the Gene Ontology term “Response to oxidative stress” in *PON1/Tub*, *+/Tub* and *+/+ D. melanogaster*. Columns represent samples and rows correspond to genes. Color scale corresponds to RMA expression value. * = genes differentially expressed between *+/Tub* and *PON1/Tub* (FDR<0.01). n = 4 aliquots of 20 flies per genotype.

PON1 has been associated to decreased oxidative stress in human tissues [13]–[18]. As an initial screen to determine whether PON1 decreases oxidative stress in *D. melanogaster*, we analyzed data from gene expression microarrays of *+/Tub* and *PON1/Tub* flies (Gene Expression Omnibus accession #GSE29534 in http://www.ncbi.nlm.nih.gov/geo/) (Figure S1). We used RMA expression values in an ANOVA model to determine fold change and false discovery rate (FDR) of gene expression between *+/Tub* and *PON1/Tub* flies. We then extracted the data from genes included in the Gene Ontology term “Response to oxidative stress” (GO:0006979; http://amigo.geneontology.org/cgi-bin/amigo/go.cgi). The heatmap in Figure 1 shows that 26 out of 45 genes in our dataset that are associated with the “Response to oxidative stress” GO Term were differentially expressed between *+/Tub* and *PON1/Tub* flies (FDR<0.01) but not between *+/Tub* and *+/+*flies (See also table S1). These data suggest that PON1 alters oxidative stress in *D. melanogaster*.

### Expression of human PON1 decreases superoxide levels in the gut of *D. melanogaster*

Regulation of ROS and the oxidative state in organisms is a complex and incompletely understood process. Screening for expression level changes of genes associated to oxidative stress in microarray data revealed that some genes are upregulated and others downregulated when PON1 is expressed in *D. melanogaster*. In order to perform mechanistic studies, we focused on the effect of PON1 expression on superoxide anion (O_2_^.−^) levels, which have been shown to play an important role in gut immunity in *D. melanogaster* [33], [34]. We hypothesized that PON1 would decrease the levels of O_2_^.−^ in *D. melanogaster*.

**Figure 2:**
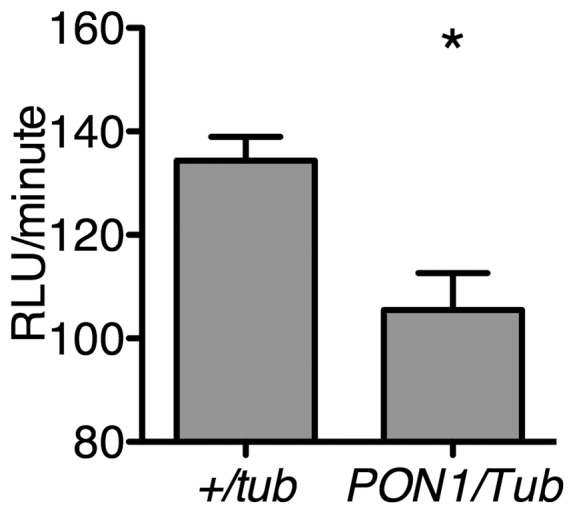
Expression of human PON1 decreases superoxide levels in *D. melanogaster*. Lucigenin staining of whole *+/Tub* and *PON1/Tub* flies. Data shown are rate of luminescence mean ± s.e.m. n = 5 pools of 3 flies per genotype. * = p<0.05, unpaired t-test.

**Figure 3:**
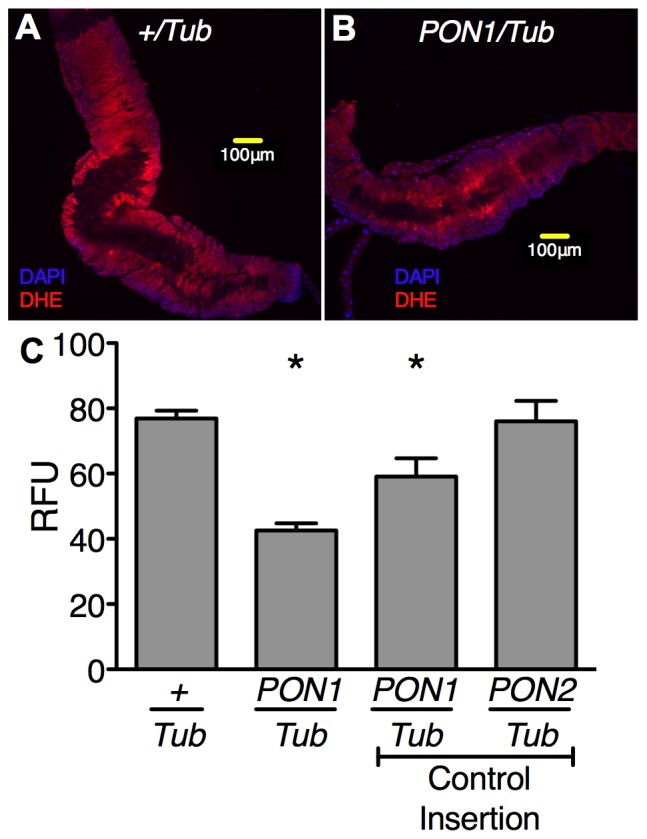
Expression of human PON1 decreases superoxide levels in the gut of *D. melanogaster*. (**A and B**) Dihydroethidium (DHE) staining and confocal microscopy were used to detect superoxide in dissected midguts from +/Tub and *PON1/Tub* flies. (**C**) Dihydroethidium staining epifluorescence intensity in midguts of *+/Tub* and *PON1/Tub* flies (P-element system insertion). Controls are *PON1/Tub and PON2/Tub* insertions using the phiC31 system. Data are mean ± s.e.m. n = 10 to 40 midguts per genotype. * = p<0.05 vs. *+/Tub*, unpaired t-test.

We used two different methods to quantify O_2_^.−^ levels in *D. melanogaster* more accurately [42]. First, we measured O_2_^.−^ levels in whole *+/Tub* and *PON1/Tub* flies using a lucigenin-based assay (Figure 2). We found that O_2_^.−^ levels were significantly lower in *PON1/Tub* compared to *+/Tub* flies. Second, we used dihydroethidium staining to directly determine O_2_^.−^ levels in the midgut of *+/Tub* and *PON1/Tub* flies. To investigate the location of O_2_^.−^, we used confocal microscopy. Figure 3 A and B shows that O_2_^.−^ can be detected with dihydroethidium in the midgut of both *+/Tub* and *PON1/Tub* flies. In order to quantitate O_2_^.−^ levels, we used epifluorescence microscopy to assess many midguts. Figure 3C shows that the intensity of dihydroethidium staining is significantly decreased in the midgut of *PON1/Tub* flies.

The method (P-element system) used to insert the *PON1* transgene into a random site of the *D. melanogaster* genome can potentially result in secondary effects due to disruption of the insertion site. We therefore included analysis of a different *D. melanogaster* line with *PON1* insertion in a different but known genomic site (phiC31 system) as a control to exclude transgene insertion site effects in *PON1/Tub* flies. We found that expression of PON1 by insertion of the transgene in the alternative site also resulted in decreased O_2_^.−^ levels. Moreover, insertion of *PON2*, which is another member of the paraoxonase family, in this same alternative site, did not result in decreased O_2_^.−^ levels (Figure 3C). Additionally, flies expressing PON1 do not have decreased longevity under standard rearing conditions (Figure S2). This results show that PON1 decreases O_2_^.−^ levels in the midgut of *D. melanogaster* and suggest that this effect is not caused by insertion site disruption or by indirect health-impairing mechanisms.

### Expression of human PON1 alters the gut microbiome of *D. melanogaster*

**Figure 4:**
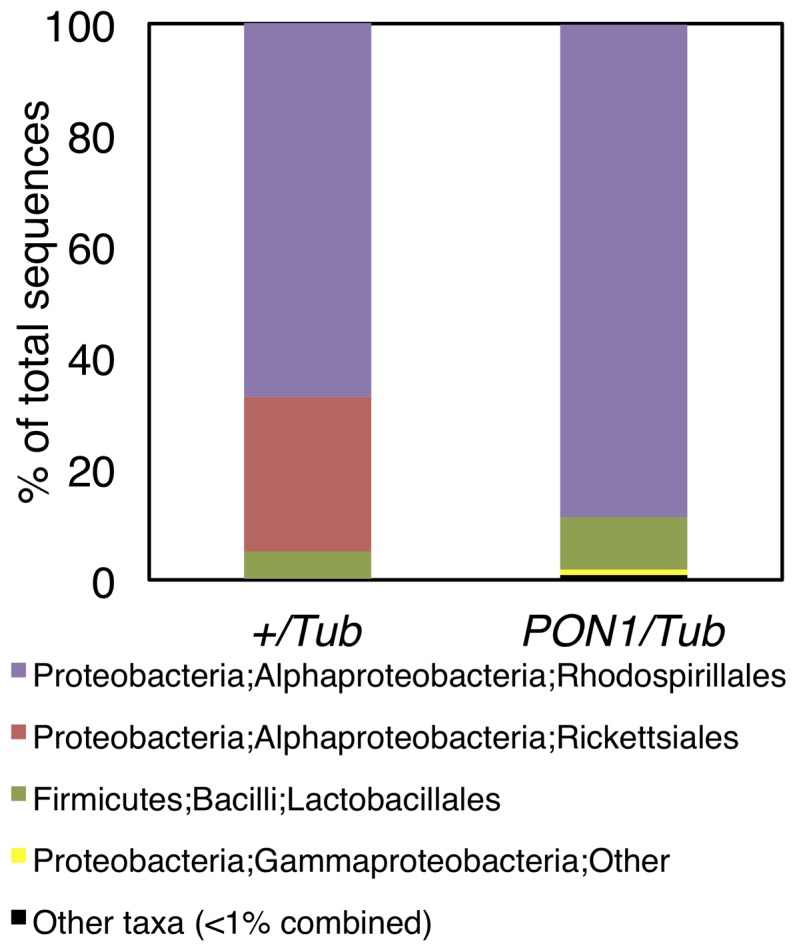
Expression of PON1 alters the composition of gut microbiota in *D. melanogaster*. High-throughput sequencing analysis of the V2 region of the bacterial 16 s rRNA gene in DNA extracted from guts of *+/Tub* and *PON1/Tub* flies. Figure shows taxonomy summary chart of order abundance as an average of 3 samples of 20 guts.

Generation of ROS by the Duox system is one of the primary mechanisms that controls gut microbial populations in *D. melanogaster* [33]–[37]. We therefore hypothesized that decreased O_2_^.−^ levels in the midgut could result in a secondary phenotype of altered colonization by midgut bacteria. We extracted genomic DNA from pools of 20 guts from +/Tub and PON1/Tub flies in triplicate and amplified a portion of the V2 region of the 16 s rRNA gene of bacteria using barcoded primers, followed by high-throughput sequencing of amplicons. We generated approximately 20,000 high quality sequences per sample. Sequences were demultiplexed and analyzed using QIIME virtual box [43]. OTU's were generated using sequences with >97% similarity, and the RDP classification method was used to assign a taxonomic identity based on the most current RDP database, to a representative sequence from each OTU. A taxonomy summary chart at the Order level was generated and the average of 3 samples per group is shown in Figure 4. 99% of the detected sequences corresponded to the orders Rhodospirillales, Rickettsiales, and Lactobacillales (with the most common species for each order being *Acetobacter aceti*, *Wolbachia endosymbiont of Drosophila melanogaster*, and *Lactobacillus plantarum*, respectively). Other Orders detected (<1% of total combined frequency) include Rhizobiales, Bacillales, Clostridiales, Sphingomonadales, Flavobacteriales and Bacteroidales and are shown in Table S2. Interestingly, while the relative abundance of Wolbachia was dramatically decreased by PON1, the proportion of Acetobacter and Lactobacillus were increased. These data show that expression of PON1 alters the composition of the gut microbiome of *D. melanogaster* and suggest that a PON1-mediated decrease in midgut O_2_^.−^ may preferentially affect specific types of bacteria.

### Expression of human PON1 increases the gut load of *Lactobacillus* in *D. melanogaster*

**Figure 5:**
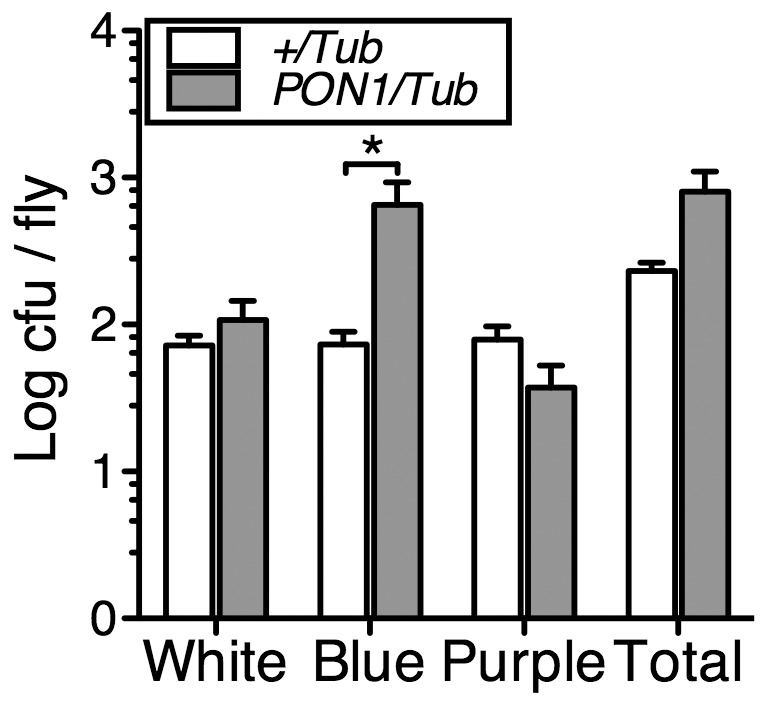
Expression of PON1 alters the composition of the culturable gut microbiota in *D. melanogaster*. Guts of *+/Tub and PON1/Tub* flies 3–5 days post eclosion were homogenized and plated on Chromagar™ Orientation plates. Number of white, blue, purple and total colonies per gut are shown, data is log average ± s.e.m . n = 15 guts. * = p<0.05, unpaired t-test.

Abundance data refer to proportions so observed changes in one bacterial group could be caused by changes in abundance of another group. We therefore studied the total load of *Wolbachia* and *Lactobacillus* independently. We hypothesized that expression of PON1 would result in alterations in the load of specific bacteria in the gut of *D. melanogaster*. We first homogenized guts of *PON/Tub* and *+/Tub* flies and used the homogenates to quantify the intracellular endosymbiont *Wolbachia* using qPCR [44]. We found that levels of Wolbachia, normalized to the single-copy *Drosophila* gene *su(F)*, were almost identical, with a +/Tub to PON/Tub expression ratio of 1.06 (95% CI = 0.005–53.71). Second, we cultured the homogenates on Chromagar™ orientation plates which allow classification of colonies according to color produced. On Chromagar™ plates, β-glucosidase-producing *Lactobacillus* should generate blue colonies. Although other bacteria were expected according to the high-throughput sequencing results, this simple scheme would allow us to discern Lactobacillus, the predominant β-glucosidase-producing species detected, from other bacteria. Interestingly, whereas the total load of culturable bacteria was similar in both genotypes (Figure 5), load of blue colonies (predominantly *Lactobacillus plantarum* as confirmed by 16 s rRNA gene sequencing) was significantly elevated in PON1 flies. Interestingly, a global gene ontology-based reanalysis of our gene expression microarray data using GOrilla (http://cbl-gorilla.cs.technion.ac.il/) [45], [46] revealed altered expression of cuticle and Imd pathway antimicrobial peptide genes (Figure S3), which are important in maintaining normal gut homeostasis and control of the intraluminal bacterial population [35], [47]–[49]. These data show that expression of PON1 alters the gut microbiome of *D. melanogaster*.

### PON1-mediated decrease in gut superoxide levels in *D. melanogaster* is independent of altered bacterial colonization

**Figure 6:**
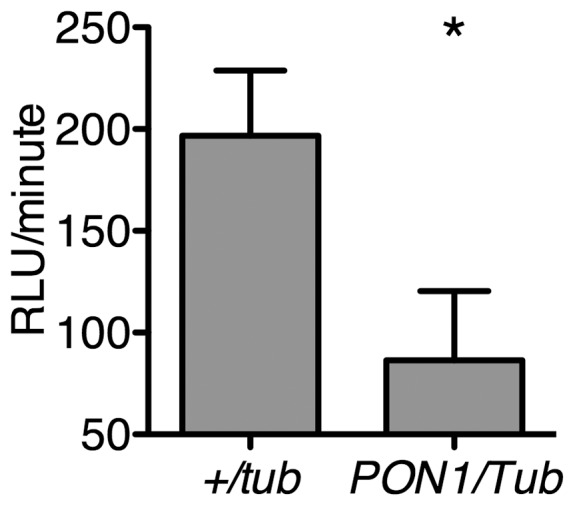
Expression of PON1 decreases superoxide levels in germ-free *D. melanogaster*. Lucigenin staining using pools of germ-free whole *+/Tub and PON1/Tub* flies. Data shown are rate of luminescence mean ± s.e.m. n = 5 pools of 3 flies per genotype. * = p<0.05, unpaired t-test.

Expression of PON1 both decreased O_2_^.−^ levels and altered the microbiome in the midgut of *D. melanogaster*. Decreased O_2_^.−^ levels could be directly caused by PON1 or could be secondary to the abnormal midgut microbiome of *PON1/Tub* flies. In order to determine whether PON1 directly decreases O_2_^.−^ levels, we generated lines of axenic (germ-free) *D. melanogaster* using the method described in [50] and compared the levels of O_2_^.−^ in *+/Tub* and *PON/Tub* flies. We predicted that in germ-free *PON/Tub* flies, levels of O_2_^.−^ would be decreased compared to *+/Tub*. Figure 6 shows that in both conventionally reared and germ-free *D. melanogaster*, PON1 expression decreases levels of O_2_^.−^. These results show that PON1 directly decreases O_2_^.−^ levels independent of changes in the midgut microbiome of *D. melanogaster*.

## Discussion

We have previously shown that human *PON1* can protect *D. melanogaster* from quorum-sensing pathogens such as *P. aeruginosa* in a physical injury model [22]. Here, we found that, even in the absence of gut bacteria, PON1 can dramatically decrease levels of O_2_^.−^ in the midgut. Expression of PON1 and the resultant decrease in correlated with marked changes in the composition of the gut microbiome, including an increased load of the symbiont *L. plantarum*.

The levels of O_2_^.−^ in the midgut of Drosophila affected the composition of the gut microbiome, but it is also possible that direct interference with bacterial quorum sensing may directly or indirectly affect the colonizing capacity of some bacterial species [51]. Interestingly, this abnormal gut microbiota composition correlated with increased expression of antimicrobial peptides, perhaps, though not proven here, because the increased load of *L. plantarum* resulted in compensatory PGRP-LC-mediated activation of the Imd pathway [52], [53]. It has previously been proposed that both the Toll and Imd pathways are not only responsive to pathogens but also to symbionts that can constitute a threat under certain circumstances [38]. Thus, an abnormal deficit in function in a mechanism of midgut bacterial control in *D. melanogaster* (ROS) may result in compensatory activation of another antimicrobial mechanism (Imd).

The pattern observed in this study highlights the bidirectional and complex nature of the interaction between host genotype and composition of the gut microbiome. Some studies have suggested that genetic and physiologic factors of the host [26], [28], [29], [54] can induce changes in the abundance of some bacterial phylotypes, while other studies have shown that specific compositions of gut bacterial communities [23], [24], [27], [55] or specific symbionts [56] can affect the immune status and metabolism of the host. Here, we show that expression of a single gene can affect the composition of gut bacteria and result in an altered immune status in the host. This suggests that a single mutation in the gene of a human host can affect host physiology in unexpected ways when this mutation affects the composition of the gut microbiome. Some potential conditions in which this effect may be observed include human gastrointestinal, cardiovascular, and metabolic diseases such as inflammatory bowel disease, atherosclerosis, and diabetes mellitus. Interestingly, mutations in PON1 have been associated with all of these conditions, and the possibility of microbiome-mediated effects in these phenotypes is intriguing.

How does PON1 affect the levels of ROS and the composition of the gut microbiome in *Drosophila*? Our study suggests that PON1 may directly affect components of the system that generates and clears ROS in the midgut, but determining which components are affected and how this results in altered gut bacterial colonization will require further characterization.

In summary, expression of PON1directly leads to a decrease in midgut O_2_^.−^ levels and abnormal oxidative stress gene expression with an associated alteration in the gut microbiome in *D. melanogaster*. These changes may then lead to an altered innate antimicrobial peptide expression profile. Mutations in PON1 or other ROS modulating genes may result in an altered gut microbiome as an intermediate mechanism for diseases such as atherosclerosis and diabetes mellitus, and may also affect important traits such as longevity. Exploring the mechanisms that link genotypes in PON and other genes to bacterial homeostasis in the gut and other organs and health in the host will likely reveal important new findings.

## Methods

### Transgenic *D. melanogaster* Lines

Drosophila stocks were reared on standard cornmeal-agar-molasses medium at room temperature (21–25°C). The tubulin-Gal4 transgenic line (y^1^ w^*^; P{tubP-GAL4}LL7/TM3, Sb^1^) was obtained from the Bloomington stock center. We used the P-element system (random insertion site) to generate the *hPON1/Tub* flies used in most experiments [57] and the PhiC31 system (site-specific integration) for insertion site control experiments [58], [59]. Human PON1 cDNA was cloned into the pUAST (P-element) vector or the pUASTattB (PhiC31) vector and subsequently injected into corresponding y w^1118^ (P-element) or 51D (PhiC31) *D. melanogaster* embryos using standard techniques (Rainbow Transgenic Flies, Inc. Camarillo, CA). The binary GAL4-UAS system and tubulin (tub) promoter were used for the ubiquitous transgenic expression of PON1. *y w^1118^; ; tub-GAL4/TM3,Sb* virgin females were crossed to the corresponding P-element or PhiC31*UAS-hPON1* transgenic males to obtain flies heterozygous for both *PON1* and *Tub*. Male w^1118^ were used for +/Tub controls. F1 progeny were tested for expression of PON1 by performing western blots using an antibody to PON1 (Abcam, Cambridge, MA).

### Axenic *D. melanogaster*

Axenic fly stocks were generated according to the method described by Bakula [50]. Approximately 20 adult males and 30 virgin females were allowed to acclimatize on orange juice plates with yeast for 24 h. at 29°C. After acclimation, embryos were collected every 12 h. Embryos were then dechorionated in sodium hypochlorite (2-fold diluted bleach) for 2 min., washed twice with 70% ethanol, and then washed twice with sterile, distilled water in a sterile tissue culture hood. Control embryos were only washed with water. Embryos were then transferred to autoclaved sterile food vials. Absence of bacteria was confirmed by culture and 16 s rRNA PCR. Flies were collected for experiments 3 days after eclosion.

### Gut dissections

The entire gastrointestinal tract was removed intact from females for staining and further processing by grasping below the head and at the tip of the abdomen with fine forceps and gently pulling.

### Dihydroethidium-based superoxide quantification

Female adult fly guts (3–5 days post-eclosion, from same parents and bottle) were dissected as previously described under 1× Schneider's Drosophila Media (Gibco, Invitrogen, Carlsbad, CA). The guts were then stained with DHE using a previously described method (http://www.nature.com/protocolexchange/protocols/414) [42], [60]. Epifluorescence imaging was performed using an Olympus IX71 epifluorescence microscope. Confocal imaging was performed with an Olympus FV1000 confocal microscope. Quantification of fluorescence was performed in ImageJ [61] v1.42 (http://rsbweb.nih.gov/ij/index.html) using mean gray area measurements of outlined whole midguts.

### Lucigenin-based superoxide quantification

To assess O_2_^.−^ levels in whole flies, a lucigenin based ROS assay was performed on fly lysate as described in [62], [63]. Fly lysates were prepared using 3-day old male whole flies. For each sample 3 flies were homogenized with a pestle in mitochondria buffer (1 mM Tris pH 7.5, 20 µM EDTA, Aprotinin 2 ng/mL, Leupeptin 2 ng/mL, Pepstatin 2 ng/mL). After sonication, lysates were centrifuged and supernatant collected. A Bradford assay was completed on all samples prior to conducting the experiment. 50 µg of protein were diluted in 1× PBS to a final volume of 1 ml. Lucigenin (5 µM) and NADPH (100 µM) (Sigma-Aldrich, St. Louis, MO) were added to each sample, and luminescence was recorded every 30 s for 10 min. Initial rate was defined as the linear slope of the data points from 30 s to 150 s.

### *D. melanogaster* Gut DNA extractions

3-day old female flies were mixed with 3-day old males and allowed to incubate on sterile corn meal food prior to dissection. Females were anesthetized with CO_2_ and decapitated. The males were discarded. Female bodies were submerged under 1× PBS +/+ and the midgut, excluding the crop, was dissected. Forceps were sterilized frequently throughout. 20 guts were dissected per sample, triplicate samples were used for each genotype. Guts were homogenized with a bead beater for 30 s in 750 µL RLT buffer, using Matrix E bead tubes (MP Biomedical, Solon, OH). DNA was extracted using a DNeasy Blood & Tissue Kit (Qiagen, Valencia, CA) and eluted into 50 µL TE buffer.

### Microbiome pipeline/analysis

DNA was processed for sequencing in a Roche GS FLX 454 Titanium Series (454 Life Sciences, Branford, CT) at the University of Iowa DNA facility. The V2 region of bacterial 16S rDNA was amplified using a FastStart High Fidelity PCR System (Roche Applied Science, Indianapolis, IN). Each 25 µL reaction contained 25 ng of column purified DNA (DNeasy Blood and Tissue Kit, Qiagen, Valencia, CA), 2.5 µL FastStart 10× Buffer, 0.25 FastStart HiFi Polymerase, 0.4 µM of a modified primer 8F (also referred to as in some publications) [5′-CCTATCCCCTGTGTGCCTTGGCAGTC- **TCAG-** *AGAGTTTGATCCTGGCTCAG*-3′; composite of GS FLX Titanium Primer B (underlined), four-base library key (TCAG), and the universal bacterial primer 8F (italics)], and 0.4 µM of a modified primer 338R [5′-CCATCTCATCCCTGCGTGTCTCCGAC- **TCAG-**NNNNNNNNNN-TGCTGCCTCCCGTAGGAGT-3′; GS FLX Titanium Primer A (underlined), a unique 10-base Extended Multiplex Identifier (Ns), four-base library key (TCAG), and the broad-range bacterial primer 338R (italics)] [28]. Primers were HPLC purified and acquired from Integrated DNA Technologies (Coralville, IA). Cycling conditions were 94°C for 3 min., followed by 30 cycles of 94°C for 1 sec., 60°C for 45 sec., and 72°C for 1 min. Size and quality of the resulting PCR products was confirmed by agarose gel electrophoresis. Replicate PCRs were pooled and amplicons purified using Ampure magnetic purification beads (Agencourt Beckman Coulter, Danvers, MA). Subsequent steps for sequencing were performed following manufacturer recommended protocols.

### Culturable microbiome of *D. melanogaster*

Male and female flies of each genotype, from the same set of parents were kept in bottles on standard cornmeal agar. At designated days post-eclosion, twenty male flies were taken from the bottles, anesthetized with CO_2_ and surface sterilized by rinsing sequentially in 70% ethanol and two fresh aliquots of 1× PBS (Gibco, Invitrogen, Carlsbad, CA). Flies were then placed in fresh refrigerated 1× PBS −/−, and ground with a pestle until thoroughly homogenized (2–3 min.). Serial dilutions were made and plated on ChromAgar™ Orientation plates (BD Biosciences, San Jose, CA) and incubated at 37°C until visible colonies grew. Colonies were counted and categorized by color. Colony PCR using 27F and 1492R primers for the 16 s rRNA gene followed by standard Sanger sequencing was used to confirm identity of blue ChromAgar colonies.

### *D. melanogaster* RNA extraction

Twenty male flies age 3–5 days and cultured in the same bottle of each genotype were selected and homogenized in TRIzol reagent (Invitrogen, Carlsbad, CA) using Lysis Matrix E tubes (MP Biomedical, Solon, OH). Samples were bead-beated for 45 sec. RNA extraction was done with the Invitrogen PureLink RNA Mini kit (Invitrogen, Carlsbad, CA), per instruction manual for extractions using the TRIzol reagent.

### Microarray hybridizations

Roche NimbleGen 12plex HD2 gene expression microarrays (Design number 080813_Dmel_exp_HX12, Roche Nimblegen, Madison, WI) were used to analyze *D. melanogaster* gene expression across 3 genotypes (*+/+, +/Tub and PON1/Tub*), providing four technical replicate hybridizations per sample. Oligo dT primers (Invitrogen, Carlsbad, CA) were used to generate double-stranded cDNA from total RNA isolated. The cDNA was then amplified/labeled using Cy3-coupled random nonamers and hybridizations were performed using 4 ug of labeled cDNA per subarray as directed by the Roche NimbleGen Gene Expression Protocol (see http://www.nimblegen.com/products/lit/expression_userguide_v5p0.pdf for specific labeling and processing details). After hybridization for 16–20 h., the arrays were washed, dried, and then scanned on an Axon GenePix 4000B microarray scanner from Molecular Devices (Sunnyvale, CA).

### Microarray analysis

Raw data .pair files passing standard quality control criteria were imported into Partek Genomics Suite v6.4 for computation of RMA expression values [64]. The gene expression calls from each of the four technical replicates per sample had R-squared values of greater than 0.97.

The data was deposited in the Gene Expression Omnibus site (http://www.ncbi.nlm.nih.gov/geo/) under GEO Accession #GSE29534 using MIAME standards. Hierarchical clustering analysis was performed in Gene Pattern (http://www.broadinstitute.org/cancer/software/genepattern/) [65]. RMA values generated in Partek were used to generate a .gct format file. The .gct file was used as the input file for the “HierarchicalClustering” module in Gene Pattern with Pearson correlation used as distance measure and Pairwise complete-linkage as clustering method. The resulting output files were used in the “HierarchicalClusteringViewer” module of Gene Pattern, and a .txt Sample Information File was used to label each sample with sample genotype. The resulting file was edited in the GNU Image Manipulation Program GIMP v 2.6 (http://www.gimp.org/) for further annotation.

ANOVA analysis was performed in Partek Genomics Suite comparing +/+, +/Tub and PON1/Tub samples. Data in the resulting spreadsheet containing p-values and log_2_ transformed fold changes was used in Prism v5.0 for Mac to generate volcano plots. Probes differentially expressed between +/+ and +/Tub samples were considered to be affected by the Tub driver insertion and were excluded from further analysis. To perform gene ontology analysis, Partek Genomics Suite was used to generate a list containing probes that pass a threshold of false discovery rate of 0.01 (1%) between +/Tub and PON/Tub samples. Probes in this spreadsheet were ranked according to fold change magnitude from highest to lowest. The list was used in GOrilla (http://cbl-gorilla.cs.technion.ac.il/) [45], [46] to generate directed acyclic graphs of the Gene Ontology terms enriched in the submitted gene list. The generated GO Term lists were filtered to include only those with at least 4 genes enriched in the GO Term. The AmiGO browser (GO database release 2012-05-05 in http://amigo.geneontology.org/cgi-bin/amigo/go.cgi) was used to obtain the list of genes in the GO term “Response to oxidative stress” (GO:0006979) and this list was crossmatched to the ANOVA spreadsheet in Partek to generate heatmaps (as described above) of oxidative stress-related genes.

### Statistical analysis

All experiments were performed at least 3 times separately. Statistical analyses were performed in Graphpad Prism 5 for MacOS X. A p-value<0.05 in unpaired t-tests was considered statistically significant [66].

## Supporting Information

Figure S1 **Expression of PON1 alters the gene expression profile of** ***D. melanogaster*** **.** Total RNA from *+/+*, *+/Tub* and *PON1/Tub* flies was extracted and analyzed on *D. melanogaster* gene expression arrays. (**A**) Unsupervised hierarchical clustering and (**B**) volcano plot. Heatmap of the 25 most highly differentially (**C**) downregulated and (**D**) upregulated genes in *PON1/Tub* compared to *+/Tub* and *+/+* flies. Color key shows corresponding normalized RMA expression values.
(TIFF)

Figure S2 **Expression of PON1 does not affect longevity of** ***D. melanogaster*** **.** Survival of *+/Tub* and *PON1/Tub* flies was followed over 60 days. Data shown are % flies alive. n = 200 flies per genotype. Curves are not statistically different using the log-rank test.
(TIFF)

Figure S3 **Expression of PON1 alters the gene expression profile of immune system-related genes in** ***D. melanogaster*** **.** Total RNA from *+/+*, *+/Tub* and *PON1/Tub* flies was extracted and analyzed on *D. melanogaster* gene expression arrays. Global gene ontology analysis in GOrilla revealed PON1-induced differential expression of groups of genes associated to gene ontology terms (**A**) sensory perception of chemical stimulus, (**B**) chitin metabolic process, (**C**) antibacterial humoral response and (**D**) structural constituent of cuticle, shown as heatmaps. Color key shows corresponding normalized expression values.
(TIFF)

Table S1 **Expression of genes associated with gene ontology term “Response to oxidative stress” in** ***PON1/Tub*** **and** ***+/Tub*** **flies.** Bold columns correspond to genes differentially express between *PON1/Tub* and *+/Tub* flies but not between control genotypes *+/Tub* vs. *+/+*.
(PDF)

Table S2 **Taxonomy summary table (order level) of gut microbiota of** ***PON1/Tub*** **and** ***+/Tub*** **flies.** Each column shows relative abundance results from a pool of 20 guts. 3 replicates per genotype are shown.
(PDF)
