# Supplementary figures and images for: Expression of Human Paraoxonase 1 Decreases Superoxide Levels and Alters Bacterial Colonization in the Gut of Drosophila melanogaster
Source: PLoS One. 2012 Aug 30;7(8):e43777. doi: 10.1371/journal.pone.0043777 (PMC3431398; doi:10.1371/journal.pone.0043777)

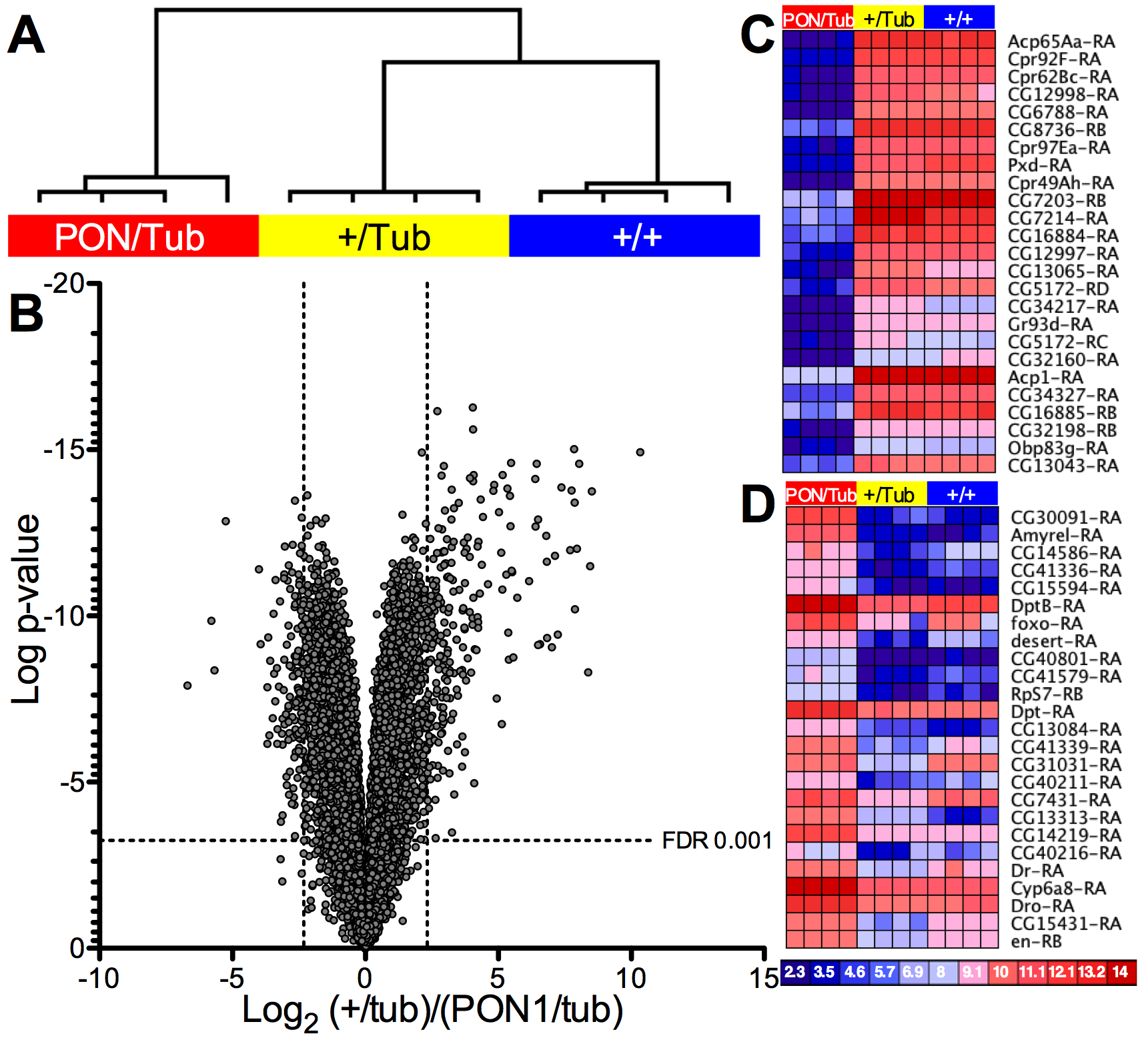

Supplement: Figure S1 — Expression of PON1 alters the gene expression profile of D. melanogaster . Total RNA from +/+, +/Tub and PON1/Tub flies was extracted and analyzed on D. melanogaster gene expression arrays. (A) Unsupervised hierarchical clustering and (B) volcano plot. Heatmap of the 25 most highly differentially (C) downregulated and (D) upregulated genes in PON1/Tub compared to +/Tub and +/+ flies. Color key shows corresponding normalized RMA expression values. (TIFF) [file pone.0043777.s001.tiff]

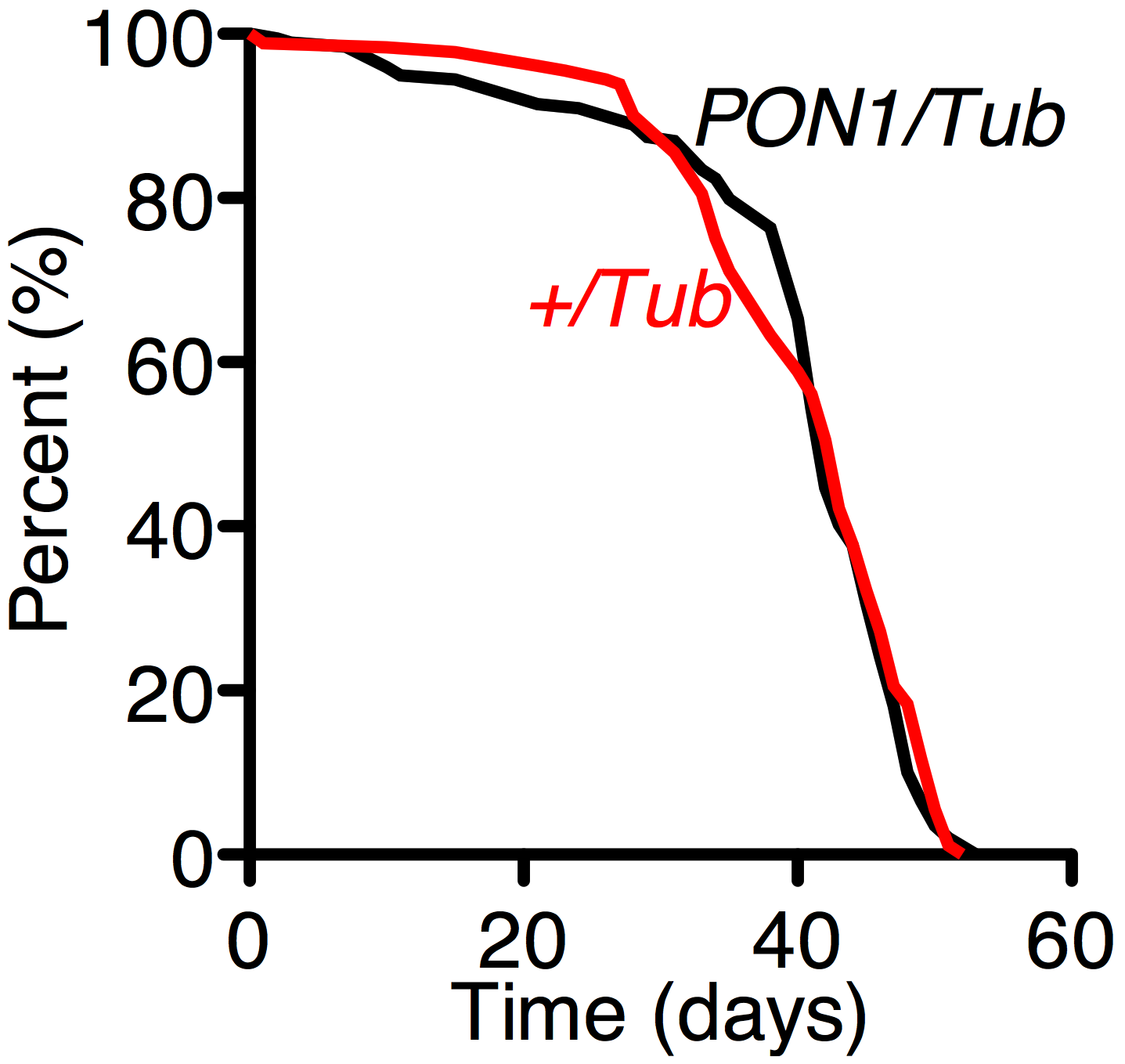

Supplement: Figure S2 — Expression of PON1 does not affect longevity of D. melanogaster . Survival of +/Tub and PON1/Tub flies was followed over 60 days. Data shown are % flies alive. n = 200 flies per genotype. Curves are not statistically different using the log-rank test. (TIFF) [file pone.0043777.s002.tiff]

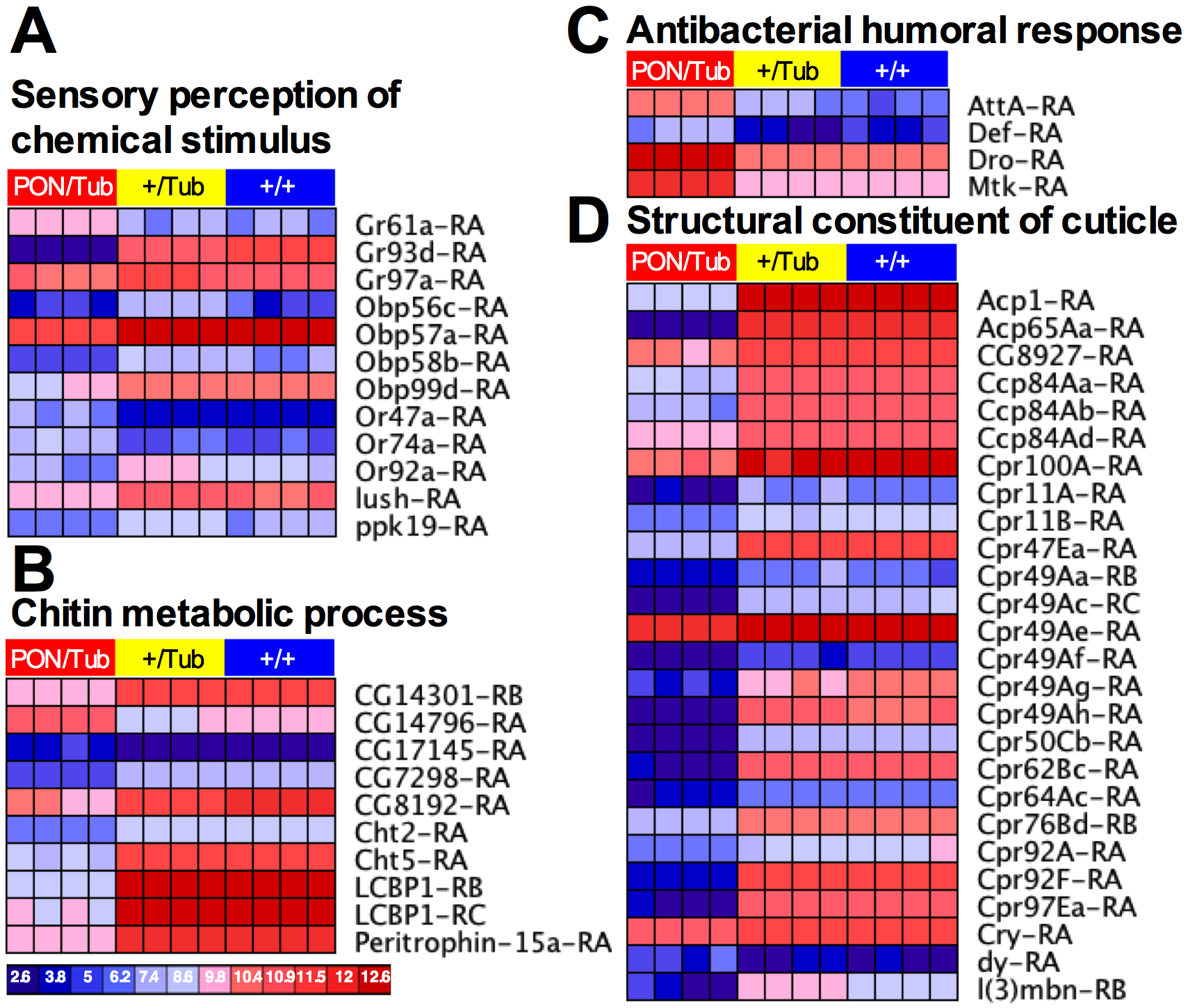

Supplement: Figure S3 — Expression of PON1 alters the gene expression profile of immune system-related genes in D. melanogaster . Total RNA from +/+, +/Tub and PON1/Tub flies was extracted and analyzed on D. melanogaster gene expression arrays. Global gene ontology analysis in GOrilla revealed PON1-induced differential expression of groups of genes associated to gene ontology terms (A) sensory perception of chemical stimulus, (B) chitin metabolic process, (C) antibacterial humoral response and (D) structural constituent of cuticle, shown as heatmaps. Color key shows corresponding normalized expression values. (TIFF) [file pone.0043777.s003.tiff]
