# Supplementary material for: Expression of Human Paraoxonase 1 Decreases Superoxide Levels and Alters Bacterial Colonization in the Gut of Drosophila melanogaster
Source: PLoS One. 2012 Aug 30;7(8):e43777. doi: 10.1371/journal.pone.0043777 (PMC3431398; doi:10.1371/journal.pone.0043777)
Supplement: Table S1 — Expression of genes associated with gene ontology term “Response to oxidative stress” in PON1/Tub and +/Tub flies. Bold columns correspond to genes differentially express between PON1/Tub and +/Tub flies but not between control genotypes +/Tub vs. +/+. (PDF) [file pone.0043777.s004.pdf]

Gene Symbol	Probeset ID	p-value (+/Tub vs. PON/tub)	Step up FDR (+/Tub vs. PON/tub)	Ratio (+/Tub vs. PON/tub)	Fold-Change (+/Tub vs. PON/tub)
alph	FBtr0085444	9.08E-05	0.000186735	0.737042	-1.35678
bsk	FBtr0080087	0.00568797	0.00833508	1.19088	1.19088
**Cat**	**FBtr0075058**	**3.30E-05**	**7.54E-05**	**0.692996**	**-1.44301**
CCS	FBtr0088424	0.0101714	0.0143126	1.16622	1.16622
**CG10211**	**FBtr0081069**	**3.47E-08**	**2.51E-07**	**1.56967**	**1.56967**
**CG15116**	**FBtr0086596**	**1.14E-09**	**2.20E-08**	**1.83528**	**1.83528**
**CG5873**	**FBtr0083442**	**1.58E-10**	**6.01E-09**	**4.99518**	**4.99518**
CG6673	FBtr0076654	7.36E-08	4.46E-07	1.51713	1.51713
CG9314	FBtr0079731	0.000306098	0.000564306	0.7807	-1.2809
Clic	FBtr0073835	0.321818	0.359888	1.04387	1.04387
cln3	FBtr0075167	0.00230024	0.00359998	0.857407	-1.16631
**cnc**	**FBtr0084392**	**4.23E-07**	**1.81E-06**	**0.543621**	**-1.83952**
CYLD	FBtr0080069	0.000644717	0.00111505	1.13143	1.13143
**DJ-1alpha**	**FBtr0087615**	**3.39E-09**	**4.58E-08**	**2.48887**	**2.48887**
**dj-1beta**	**FBtr0085703**	**1.69E-05**	**4.19E-05**	**1.41266**	**1.41266**
**Duox**	**FBtr0077724**	**7.18E-05**	**0.00015084**	**1.45571**	**1.45571**
**Egm**	**FBtr0088090**	**3.37E-05**	**7.67E-05**	**0.686296**	**-1.4571**
GstE1	FBtr0086669	0.0141643	0.0194974	1.15643	1.15643
**GstS1**	**FBtr0087005**	**9.66E-08**	**5.55E-07**	**1.8206**	**1.8206**
hang	FBtr0290069	0.15589	0.183352	1.06025	1.06025
IP3K1	FBtr0079869	0.124721	0.148604	1.05254	1.05254
Irc	FBtr0083371	0.136128	0.161444	0.91699	-1.09052
**Itp-r83A**	**FBtr0078686**	**1.58E-05**	**3.93E-05**	**0.717955**	**-1.39284**
Keap1	FBtr0083422	0.0514593	0.0652875	0.912244	-1.0962
**Mekk1**	**FBtr0083683**	**0.000246003**	**0.000461287**	**0.780893**	**-1.28058**
**Mnn1**	**FBtr0079402**	**1.34E-09**	**2.46E-08**	**0.31964**	**-3.12852**
mol	FBtr0080677	0.000229299	0.00043299	1.14674	1.14674
**Mt2**	**FBtr0110911**	**2.62E-05**	**6.16E-05**	**0.504181**	**-1.98342**
**mth**	**FBtr0072571**	**6.98E-07**	**2.73E-06**	**2.25173**	**2.25173**
**Nf1**	**FBtr0100113**	**5.79E-10**	**1.40E-08**	**0.444715**	**-2.24863**
**NLaz**	**FBtr0077942**	**1.12E-09**	**2.19E-08**	**1.76558**	**1.76558**
park	FBtr0078318	0.0853405	0.104566	0.940813	-1.06291
**per**	**FBtr0070477**	**1.43E-07**	**7.53E-07**	**0.449621**	**-2.2241**
**Pi3K92E**	**FBtr0083940**	**5.35E-09**	**6.26E-08**	**0.655721**	**-1.52504**
**Pink1**	**FBtr0070956**	**0.000158029**	**0.000308928**	**0.667517**	**-1.49809**
puc	FBtr0081792	1.10E-06	4.00E-06	0.760439	-1.31503
**Pxd**	**FBtr0089287**	**3.90E-14**	**2.30E-11**	**234.295**	**234.295**
Pxn	FBtr0072951	0.000444064	0.000791551	0.791308	-1.26373
**Pxt**	**FBtr0083508**	**0.00651843**	**0.00945047**	**0.659348**	**-1.51665**
**rut**	**FBtr0073992**	**4.22E-06**	**1.25E-05**	**0.534901**	**-1.8695**
Sod	FBtr0076229	0.000958457	0.00160819	1.14677	1.14677
**sun**	**FBtr0074177**	**1.88E-10**	**6.69E-09**	**3.66959**	**3.66959**
Thor	FBtr0077524	0.000155494	0.000304366	1.19811	1.19811
**TORC**	**FBtr0075196**	**2.27E-09**	**3.48E-08**	**0.395293**	**-2.52977**
**Trx-2**	**FBtr0079839**	**5.77E-07**	**2.33E-06**	**1.48506**	**1.48506**

Supplemental Table S1. Expression of genes associated with gene ontology term “Response to oxidative stress” in *PON1/Tub* and *+/Tub* flies. Bold columns correspond to genes differentially express between *PON1/Tub* and *+/Tub* flies but not between control genotypes *+/Tub* vs. *+/+*.
