# Supplementary material for: Expression of Human Paraoxonase 1 Decreases Superoxide Levels and Alters Bacterial Colonization in the Gut of Drosophila melanogaster
Source: PLoS One. 2012 Aug 30;7(8):e43777. doi: 10.1371/journal.pone.0043777 (PMC3431398; doi:10.1371/journal.pone.0043777)
Supplement: Table S2 — Taxonomy summary table (order level) of gut microbiota of PON1/Tub and +/Tub flies. Each column shows relative abundance results from a pool of 20 guts. 3 replicates per genotype are shown. (PDF) [file pone.0043777.s005.pdf]

| Taxon | *PON/Tub-1* | *PON/Tub-2* | *PON/Tub-3* | *+/Tub-1* | *+/Tub-2* | *+/Tub-3* |
|---|---|---|---|---|---|---|
| Root;Bacteria;Proteobacteria;Alphaproteobacteria;Rhodospirillales | 0.999030138 | 0.933641346 | 0.722855857 | 0.76285544 | 0.97543378 | 0.274242175 |
| Root;Bacteria;Proteobacteria;Alphaproteobacteria;Rickettsiales | 0 | 0 | 0.000525249 | 0.105227311 | 0.019392532 | 0.704927918 |
| Root;Bacteria;Firmicutes;"Bacilli";"Lactobacillales" | 0.000826179 | 0.062417901 | 0.224506641 | 0.126809127 | 0.004188224 | 0.016800965 |
| Root;Bacteria;Proteobacteria;Gammaproteobacteria;Other | 0 | 0 | 0.025737225 | 0 | 3.52E-05 | 0.000137039 |
| Root;Bacteria;Other;Other;Other | 3.59E-05 | 0.001404176 | 0.006340512 | 0.000808786 | 0.000105585 | 0.000575563 |
| Root;Bacteria;Firmicutes;"Bacilli";Bacillales | 0 | 0.00045296 | 0.004502139 | 0.000638515 | 3.52E-05 | 0.000685194 |
| Root;Bacteria;Proteobacteria;Alphaproteobacteria;Rhizobiales | 0 | 0.000770032 | 0.002701283 | 0.000851354 | 0.000140781 | 0.000630379 |
| Root;Bacteria;Proteobacteria;Betaproteobacteria;Burkholderiales | 0 | 0.000271776 | 0.001988445 | 0.000893921 | 0 | 0.000548155 |
| Root;Bacteria;Actinobacteria;Actinobacteria;Actinobacteridae | 7.18E-05 | 4.53E-05 | 0.002138516 | 0.000595948 | 7.04E-05 | 0.000274078 |
| Root;Bacteria;Firmicutes;"Clostridia";Clostridiales | 0 | 0 | 0.002326105 | 8.51E-05 | 0.000105585 | 5.48E-05 |
| Root;Bacteria;Proteobacteria;Other;Other | 0 | 0.00045296 | 0.00086291 | 0 | 0.000281561 | 0.000137039 |
| Root;Bacteria;Firmicutes;Other;Other | 3.59E-05 | 9.06E-05 | 0.001012981 | 0.000170271 | 3.52E-05 | 0 |
| Root;Bacteria;Cyanobacteria;Cyanobacteria;Chloroplast | 0 | 0.000317072 | 0.000600285 | 0.000127703 | 3.52E-05 | 8.22E-05 |
| Root;Bacteria;Bacteroidetes;Flavobacteria;Flavobacteriales | 0 | 0 | 0.000525249 | 0.000170271 | 0 | 0.000191854 |
| Root;Bacteria;Proteobacteria;Alphaproteobacteria;Sphingomonadales | 0 | 4.53E-05 | 0.000412696 | 0.000297974 | 0 | 8.22E-05 |
| Root;Bacteria;Bacteroidetes;Other;Other | 0 | 0 | 0.000450214 | 0 | 0 | 8.22E-05 |
| Root;Bacteria;Proteobacteria;Alphaproteobacteria;Other | 0 | 0 | 0.000112553 | 4.26E-05 | 0 | 0.000219262 |
| Root;Bacteria;Proteobacteria;Betaproteobacteria;Other | 0 | 0 | 0.000262625 | 4.26E-05 | 0 | 2.74E-05 |
| Root;Bacteria;Proteobacteria;Gammaproteobacteria;Pseudomonadales | 0 | 4.53E-05 | 0.000262625 | 0 | 0 | 0 |
| Root;Bacteria;Acidobacteria;Acidobacteria;Acidobacteriales | 0 | 0 | 0.000225107 | 0 | 0 | 8.22E-05 |
| Root;Bacteria;Bacteroidetes;Bacteroidetes;Bacteroidales | 0 | 4.53E-05 | 0.000187589 | 0 | 7.04E-05 | 0 |
| Root;Bacteria;Firmicutes;"Clostridia";Other | 0 | 0 | 0.000262625 | 0 | 0 | 0 |
| Root;Bacteria;Proteobacteria;Deltaproteobacteria;Other | 0 | 0 | 0.000225107 | 0 | 0 | 0 |
| Root;Bacteria;Proteobacteria;Betaproteobacteria;Rhodocyclales | 0 | 0 | 0.000187589 | 0 | 0 | 0 |
| Root;Bacteria;Chloroflexi;Chloroflexi;Chloroflexales | 0 | 0 | 7.50E-05 | 8.51E-05 | 0 | 0 |
| Root;Bacteria;Bacteroidetes;Sphingobacteria;Sphingobacteriales | 0 | 0 | 0.000150071 | 0 | 0 | 0 |
| Root;Bacteria;Fusobacteria;Fusobacteria;Fusobacteriales | 0 | 0 | 0.000150071 | 0 | 0 | 0 |
| Root;Bacteria;Actinobacteria;Actinobacteria;Rubrobacteridae | 0 | 0 | 3.75E-05 | 0 | 0 | 0.000109631 |
| Root;Bacteria;Proteobacteria;Gammaproteobacteria;Xanthomonadales | 0 | 0 | 7.50E-05 | 0 | 3.52E-05 | 2.74E-05 |
| Root;Bacteria;Firmicutes;"Bacilli";Other | 0 | 0 | 0 | 8.51E-05 | 3.52E-05 | 0 |
| Root;Bacteria;TM7;TM7_genera_incertae_sedis;Other | 0 | 0 | 7.50E-05 | 4.26E-05 | 0 | 0 |
| Root;Bacteria;Proteobacteria;Deltaproteobacteria;Desulfovibrionales | 0 | 0 | 7.50E-05 | 0 | 0 | 0 |
| Root;Bacteria;Proteobacteria;Gammaproteobacteria;Enterobacteriales | 0 | 0 | 3.75E-05 | 0 | 0 | 2.74E-05 |
| Root;Bacteria;Deinococcus-Thermus;Deinococci;Deinococcales | 0 | 0 | 0 | 4.26E-05 | 0 | 0 |
| Root;Bacteria;Proteobacteria;Alphaproteobacteria;Caulobacterales | 0 | 0 | 0 | 4.26E-05 | 0 | 0 |
| Root;Bacteria;Cyanobacteria;Cyanobacteria;Family X | 0 | 0 | 0 | 4.26E-05 | 0 | 0 |
| Root;Bacteria;Proteobacteria;Epsilonproteobacteria;Campylobacterales | 0 | 0 | 0 | 4.26E-05 | 0 | 0 |
| Root;Bacteria;Proteobacteria;Gammaproteobacteria;Legionellales | 0 | 0 | 3.75E-05 | 0 | 0 | 0 |
| Root;Other;Other;Other;Other | 0 | 0 | 3.75E-05 | 0 | 0 | 0 |
| Root;Bacteria;Cyanobacteria;Cyanobacteria;Other | 0 | 0 | 3.75E-05 | 0 | 0 | 0 |
| Root;Bacteria;Actinobacteria;Actinobacteria;Other | 0 | 0 | 0 | 0 | 0 | 2.74E-05 |
| Root;Bacteria;Proteobacteria;Deltaproteobacteria;Bdellovibrionales | 0 | 0 | 0 | 0 | 0 | 2.74E-05 |

**Supplemental Table S2.** Taxonomy summary table (order level) of gut microbiota of *PON1/Tub* and *+/Tub* flies. Each column shows relative abundance results from a pool of 20 guts. 3 replicates per genotype are shown.
